# Histone deacetylase Sir2 promotes the systemic *Candida albicans* infection by facilitating its immune escape via remodeling the cell wall and maintaining the metabolic activity

**DOI:** 10.1128/mbio.00445-24

**Published:** 2024-04-29

**Authors:** Chen Yang, Guanglin Li, Qiyue Zhang, Wenhui Bai, Qingiqng Li, Peipei Zhang, Jiye Zhang

**Affiliations:** 1 School of Pharmacy, Health Science Center, Xi’an Jiaotong University, Xi'an, Shaanxi, China; 2 Institute of Pharmaceutical Science and Technology, Xi’an Jiaotong University, Xi'an, Shaanxi, China; Universidad de Cordoba, Cordoba, Spain

**Keywords:** *C. albicans* pathogenicity, histone deacetylase Sir2, cell wall remodeling, adhesion, β-glucan, mannan, immune evasion

## Abstract

Histone deacetylation affects *Candida albicans* (*C. albicans*) pathogenicity by modulating virulence factor expression and DNA damage. The histone deacetylase Sir2 is associated with *C. albicans* plasticity and maintains genome stability to help *C. albicans* adapt to various environmental niches. However, whether Sir2-mediated chromatin modification affects *C. albicans* virulence is unclear. The purpose of our study was to investigate the effect of Sir2 on *C. albicans* pathogenicity and regulation. Here, we report that Sir2 is required for *C. albicans* pathogenicity, as its deletion affects the survival rate, fungal burden in different organs and the extent of tissue damage in a mouse model of disseminated candidiasis. We evaluated the impact of Sir2 on *C. albicans* virulence factors and revealed that the Sir2 null mutant had an impaired ability to adhere to host cells and was more easily recognized by the innate immune system. Comprehensive analysis revealed that the disruption of *C. albicans* adhesion was due to a decrease in cell surface hydrophobicity rather than the differential expression of adhesion genes on the cell wall. In addition, Sir2 affects the distribution and exposure of mannan and β-glucan on the cell wall, indicating that Sir2 plays a role in preventing the immune system from recognizing *C. albicans*. Interestingly, our results also indicated that Sir2 helps *C. albicans* maintain metabolic activity under hypoxic conditions, suggesting that Sir2 contributes to *C. albicans* colonization at hypoxic sites. In conclusion, our findings provide detailed insights into antifungal targets and a useful foundation for the development of antifungal drugs.

**IMPORTANCE:**

*Candida albicans* (*C. albicans*) is the most common opportunistic fungal pathogen and can cause various superficial infections and even life-threatening systemic infections. To successfully propagate infection, this organism relies on the ability to express virulence-associated factors and escape host immunity. In this study, we demonstrated that the histone deacetylase Sir2 helps *C. albicans* adhere to host cells and escape host immunity by mediating cell wall remodeling; as a result, *C. albicans* successfully colonized and invaded the host *in vivo*. In addition, we found that Sir2 contributes to carbon utilization under hypoxic conditions, suggesting that Sir2 is important for *C. albicans* survival and the establishment of infection in hypoxic environments. In summary, we investigated the role of Sir2 in regulating *C. albicans* pathogenicity in detail; these findings provide a potential target for the development of antifungal drugs.

## INTRODUCTION

*Candida albicans* is a commensal pathogen that lives in the oral cavity, gastrointestinal tract, and genital regions of healthy individuals but causes life-threatening infections in immunocompromised patients (1–4). The establishment of successful infection in diverse hosts requires a wide range of virulence factors and the ability to adapt to the various environmental niches (3, 5). The transcriptional regulation of virulence factors and fitness has been extensively studied (6–8); chromatin modification also regulates the commensal and pathogenic attributes of *C. albicans* (9–11). However, unlike widely studied transcriptional regulation, the role of chromatin modulation in influencing pathogenicity patterns has not been elucidated.

Histone deacetylases (HDACs) are chromatin modulators that play crucial roles in chromatin-mediated regulation of *C. albicans* pathogenicity by directly altering morphology (10). Previous studies have confirmed that Rpd31, a class I HDAC, is required for the activation of filamentous growth (12, 13). Hda1, a class II HDAC, is also necessary for hyphal development in *C. albicans* and is responsible for hyphal elongation and maintenance (13–15). Furthermore, deletion of either RPD31 or HDA1 significantly reduced *C. albicans* pathogenicity in a mouse model (16). The class III HDACs are strongly associated with morphology transition. The sirtuin family comprises class III HDACs. The sirtuins Hst3 and Sir2 were identified as morphology switch repressors (17, 18). Interestingly, although HST3 repression causes the production of key virulence factors, *C. albicans* cell viability decreases *in vitro*, and pathogenicity decreases significantly *in vivo* due to the fragmentation and degradation of chromosomal DNA (19). Similar to Hst3, Sir2 controls the chromosome stability by protecting genes in ribosomal genomic regions from fragmentation (20). However, Sir2 does not affect the cell viability of *C. albicans* cells *in vitro* (21), and its effect on *C. albicans* pathogenicity *in vivo* remains poorly understood. Sir2 is localized to the nucleus and is specifically focused in the nucleolus, suggesting that Sir2 plays a role in regulating gene expression (22). Because Sir2-mediated genomic stability can be altered by environmental conditions, regulating relevant gene expression is an effective strategy for adapting to various niches (20). Therefore, we speculated that Sir2 may regulate the expression of virulence genes to help *C. albicans* colonize the host.

Sirtuins can sense environmental changes that alter the cell’s metabolic state (22). There is evidence that Sir2 interacts with N-acetylglucosamine kinase 1 (Hxk1) (18), which is involved in the disruption of the N-acetylglucosamine (GlcNAc) catabolic pathway, and attenuates *C. albicans* virulence *in vivo* (23). Based on these reports, we speculated that Sir2 was involved in the regulation of *C. albicans* pathogenicity by modulating metabolic activity. Furthermore, Hxk1 is involved in cell wall synthesis (24). Consequently, Sir2 may also influence the cell wall components of *C. albicans*. The cell wall is important for *C. albicans* survival *in vivo* and promotes adaptation to the environment. Pathogen-associated molecular patterns (PAMPs) in the cell wall, such as mannan, β-glucan, and chitin, are recognized by pattern recognition receptors on the surface of host cells (25–27). Among them, the recognition of mannan and β-glucan by C-type lectin receptors triggers phagocytosis and killing by neutrophils and macrophages, whereas chitin has been found to block the recognition of *C. albicans* yeast cells by monocytes (3). Therefore, changes in the cell wall polysaccharide composition directly affect immunogenicity. In addition to polysaccharides, proteins are the other main components of the *C. albicans* cell wall. These proteins are heavily mannosylated through O- and N-chemical bonds to form mannoproteins that are located in the outermost layer of the cell wall and are involved in adhesion, drug resistance, and *C. albicans* virulence (28–30). Therefore, we hypothesized that Sir2 might be involved in *C. albicans* immunomodulation and adhesion through the regulation of cell wall components.

Based on these findings, we analyzed whether Sir2 contributes to *C. albicans* pathogenicity and sought to determine how Sir2 regulates virulence. Here, we comprehensively demonstrated that Sir2 was necessary for *C. albicans* pathogenicity in a mouse model of disseminated candidiasis. We also showed that Sir2 is involved in regulating *C. albicans*-host interactions by mediating cell wall remodeling and hypoxia-related metabolic activity, thus affecting the colonization and invasion of *C. albicans*.

## RESULTS

### Sir2 is required for *C. albicans* virulence in mouse model of hematogenously disseminated candidiasis

**Fig 1:**
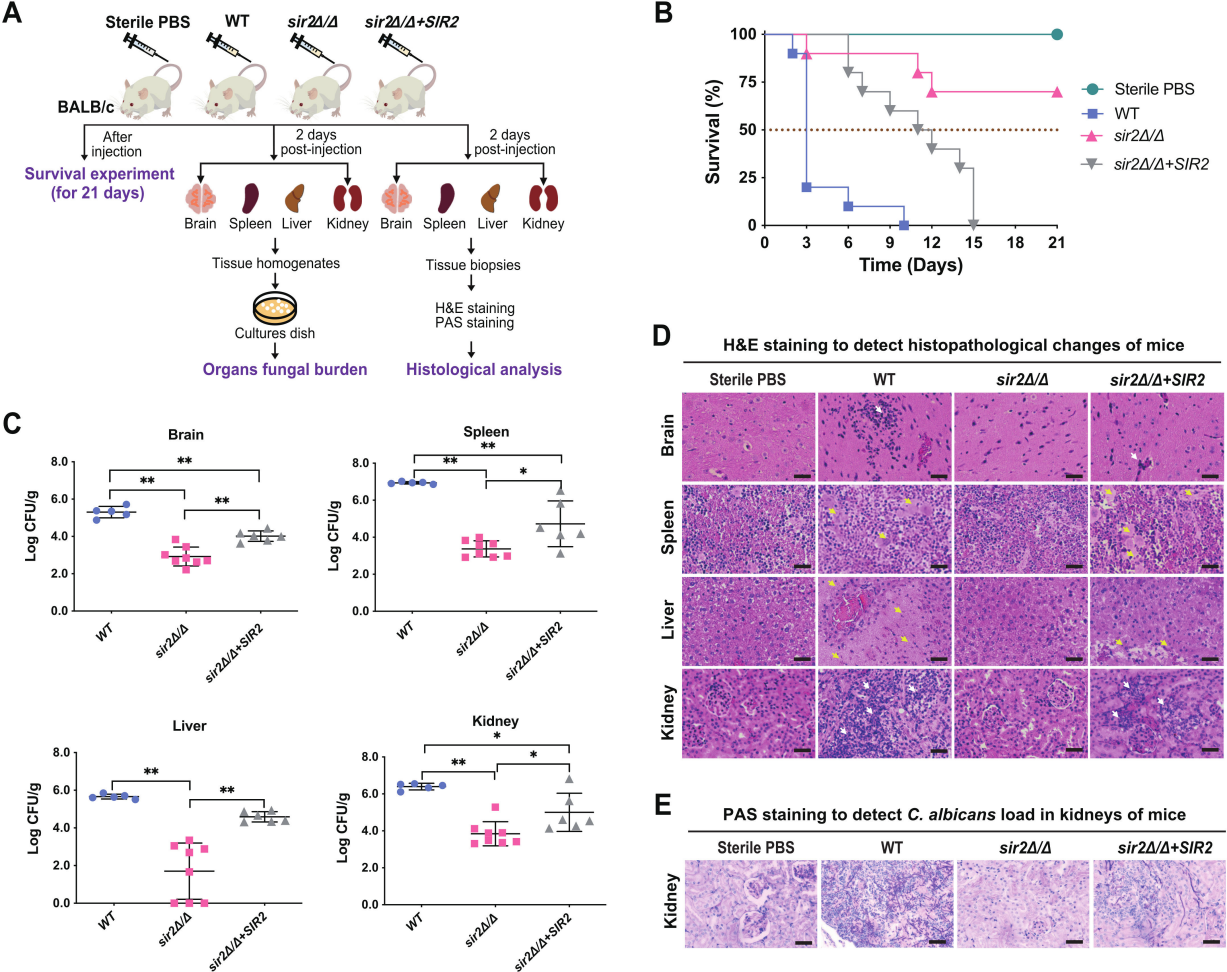
Sir2 is required for the *C. albicans* pathogenicity. (A) Schematic diagram on the overall design in the effect of Sir2 on the virulence of *C. albicans*. (B) Animal survival assays. Mice (*n* = 10) were intravenously inoculated with various *C. albicans* strains and were assessed for survival for up to 21 days post-infection. Sterile PBS as control, gray dotted line: median survival. (C) Organ fungal burden assays. Mice injected with the different strains were detected at 2 days post-infection; CFUs from brains, spleens, kidneys, and livers were assayed and plotted using scatter diagram, with error bar. Asterisks show statistically significant differences (^*^, *P* < 0.05; ^**^, *P* < 0.01) based on multiple unpaired *t* test (WT vs *sir2*Δ/Δ, WT vs *sir2*Δ/Δ+*SIR2*, and *sir2*Δ/Δ vs *sir2*Δ/Δ+*SIR2*). (D) Histopathological examination. Histopathological changes in brains, spleens, livers, and kidneys of mice were detected at 2 days post-infection by various *C. albicans* strains (hematoxylin and eosin [H&E] staining). Sterile PBS as control, white arrow: inflammatory infiltration, yellow arrow: tissue injury, bar: 20 µm. (E) Distribution of *C. albicans* in the kidneys of mice was detected at 2 days post-infection by various *C. albicans* strains (periodic acid-schiff [PAS] staining). Sterile PBS as control, bar: 50 µm.

To evaluate the role of Sir2 in *C. albicans* pathogenicity, the virulence of the *sir2*∆/∆ null mutant created by CRISPR/Cas9 (Fig. S1 and S3) was tested in a murine model of disseminated candidiasis (Fig. 1A). The wild-type (WT) *C. albicans* and *sir2*∆/∆*+SIR2* complemented strains with the *SIR2* ORF under the control of the *C. albicans* MET3 promoter were used as controls (Fig. S2 and S3). Over a 21-day observation period (Fig. 1B), three of the mice infected by intravenous tail vein injection with *sir2*∆/∆ died. In contrast, all of the mice infected with WT or *sir2*∆/∆*+SIR2* died, and the median survival times were 3 and 11 days, respectively. To evaluate fungal dissemination and colonization, the organ fungal burden was detected 2 days post-infection (Fig. 1C). The fungal burdens in all tested organs of mice inoculated with *sir2*∆/∆ were significantly lower than those in mice infected with either WT or *sir2*∆/∆*+SIR2*. Furthermore, the number of mice in each group was different because a percentage of the mice infected with either WT or the *sir2*∆/∆*+SIR2* died of infection at certain time points. Hematoxylin and eosin (H&E) staining provides a comprehensive picture of tissues (Fig. 1D). The organs of mice infected with either WT or *sir2*∆/∆*+SIR2* presented with large patchy necrosis and severe inflammatory cell infiltration, while no obvious damage was observed in the *sir2*∆/∆ and sterile PBS groups. PAS staining verified that the inflammation and damage in the kidneys of mice infected with WT or *sir2*∆/∆*+SIR2* were caused by the organism (Fig. 1E). In conclusion, Sir2 contributes to the colonization and invasion of *C. albicans* in mice.

Notably, in the fungal burden and survival experiments, the *sir2*∆/∆*+SIR2* strain did not recapitulate the WT phenotype. This may be because *SIR2* expression in the *sir2*∆/∆*+SIR2* strain does not occur under its own promoter. Thus, we constructed another complemented strain expressing *SIR2* under the control of the *SIR2* promoter (*sir2*∆/∆*+nSIR2*) (Fig. S2 and S3) and evaluated the virulence of this strain in mice. Survival, fungal burden, and histopathology confirmed that the virulence of *sir2*∆/∆+*nSIR2* returned to that of WT (Fig. S4).

### Sir2 helps *C. albicans* adhere to host cells

**Fig 2:**
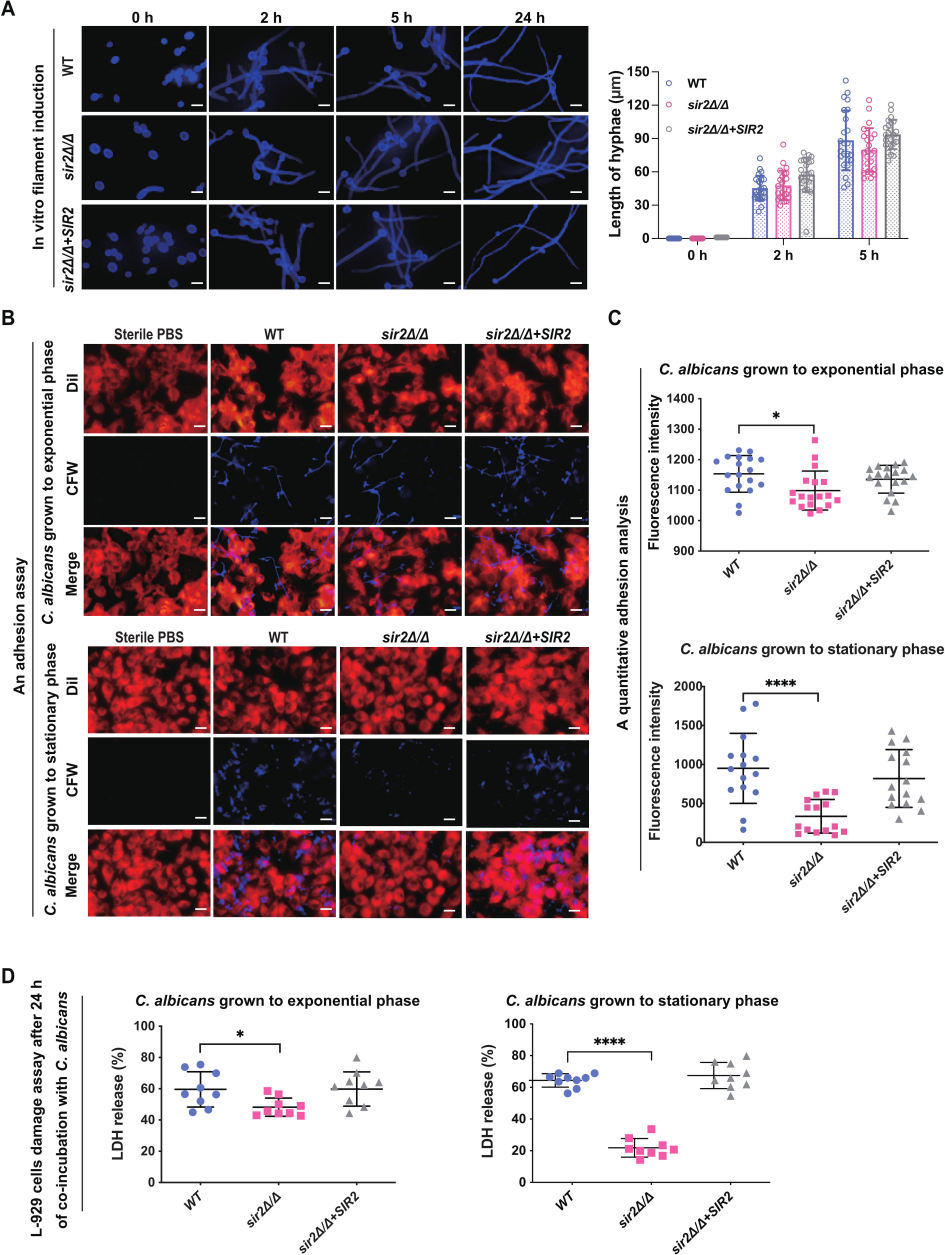
Sir2 helps *C. albicans* adhere to and invade host cells. (A) Fluorescence micrographs (left) and statistical results of hyphal length quantification (right) of various *C. albicans* strains at different time points cultured at 37°C in 1640 medium containing 10% serum; *C. albicans* stained by calcofluor white (CFW), bar: 10 µm. (B) Fluorescence micrographs of *C. albicans* adhesion on L-929 cells at 2 h (yeast-form *C. albicans* grown to exponential growth phase or stationary growth phase were co-incubated with L-929 cells). Sterile PBS as control, DiI: staining L-929 cells, CFW: staining *C. albicans*, bar: 25 µm. (C) Quantitative analysis of adhesion of *C. albicans* on L-929 cells at 2 h (yeast-form *C. albicans* grown to exponential growth phase or stationary growth phase were co-incubated with L-929 cells). The data represent the average of three independent replicates, with error bars. Asterisks show statistically significant differences (^*^, *P* < 0.05; ^****^, *P* < 0.001) based on one-way ANOVA (WT vs *sir2*∆*/*∆, WT vs *sir2*∆*/*∆+*SIR2*, and *sir2*∆*/*∆ vs *sir2*∆*/*∆+*SIR2*). (D) LDH release from L-929 cells infected with various *C. albicans* for 24 h. The data represent the average of three independent replicates, with error bars. Asterisks show statistically significant differences (^*^, *P* < 0.05; ^****^, *P* < 0.001) based on one-way ANOVA (WT vs *sir2*∆*/*∆, WT vs *sir2*∆*/*∆+*SIR2*, and *sir2*∆*/*∆ vs *sir2*∆*/*∆+*SIR2*).

The morphogenetic conversion of *C. albicans* from yeast to hyphal form more easily causes invasion of host tissues (31). In a mouse model of disseminated candidiasis, no obvious hyphae were observed in the kidneys of mice infected with *sir2*∆/∆ (Fig. 1E). Thus, we tested the influence of Sir2 on the morphological transition of *C. albicans* under hyphal-inducing conditions *in vitro*. In CFW-stained images, we found that neither the ratio of yeast-phase nor the hyphal length significantly differed at the observation time points among the tested strains (Fig. S5 and Fig. 2A). There were also no differences in the expression of the genes associated with hyphal development (Fig S6). Therefore, we propose that the reduced virulence of *sir2*∆/∆ in the mouse model is not due to a defect in hyphae.

In the process of colonization and infection, *C. albicans* must adhere to host cells (32). For this reason, we evaluated *C. albicans* adhesion in a coculture model using *C. albicans*-host cells (L-929 cells). Fluorescence micrographs and quantitative analysis revealed that the adhesion of *sir2*∆/∆ to L-929 cells significantly decreased compared to that of WT or *sir2*∆/∆*+SIR2* cells, and the lack of *sir2*∆/∆ adhesion was particularly pronounced when the mutant was grown to stationary phase (Fig. 2B and C ; Fig. S7). In addition, the morphologies of the strains were consistent at each observation point, which also supports our previous hypothesis that the discrepancy in tissue damage in the mouse model of disseminated candidiasis is not caused by morphological differences. Moreover, the damage to host cells in the coculture model was evaluated by measuring LDH release. LDH release from L-929 cells cocultured with *sir2*∆/∆ was significantly lower than that from L-929 cells cocultured with either WT or *sir2*∆/∆*+SIR2* (Fig. 2D). By comparing the adherence of *C. albicans* and damage to L-929 cells in each experimental group, we found that they were positively correlated, indicating that the discrepant adhesion ability of each strain was an important reason for the differences in damage to host cells.

### Sir2 mediates *C. albicans* adhesion by regulating cell surface hydrophobicity

**Fig 3:**
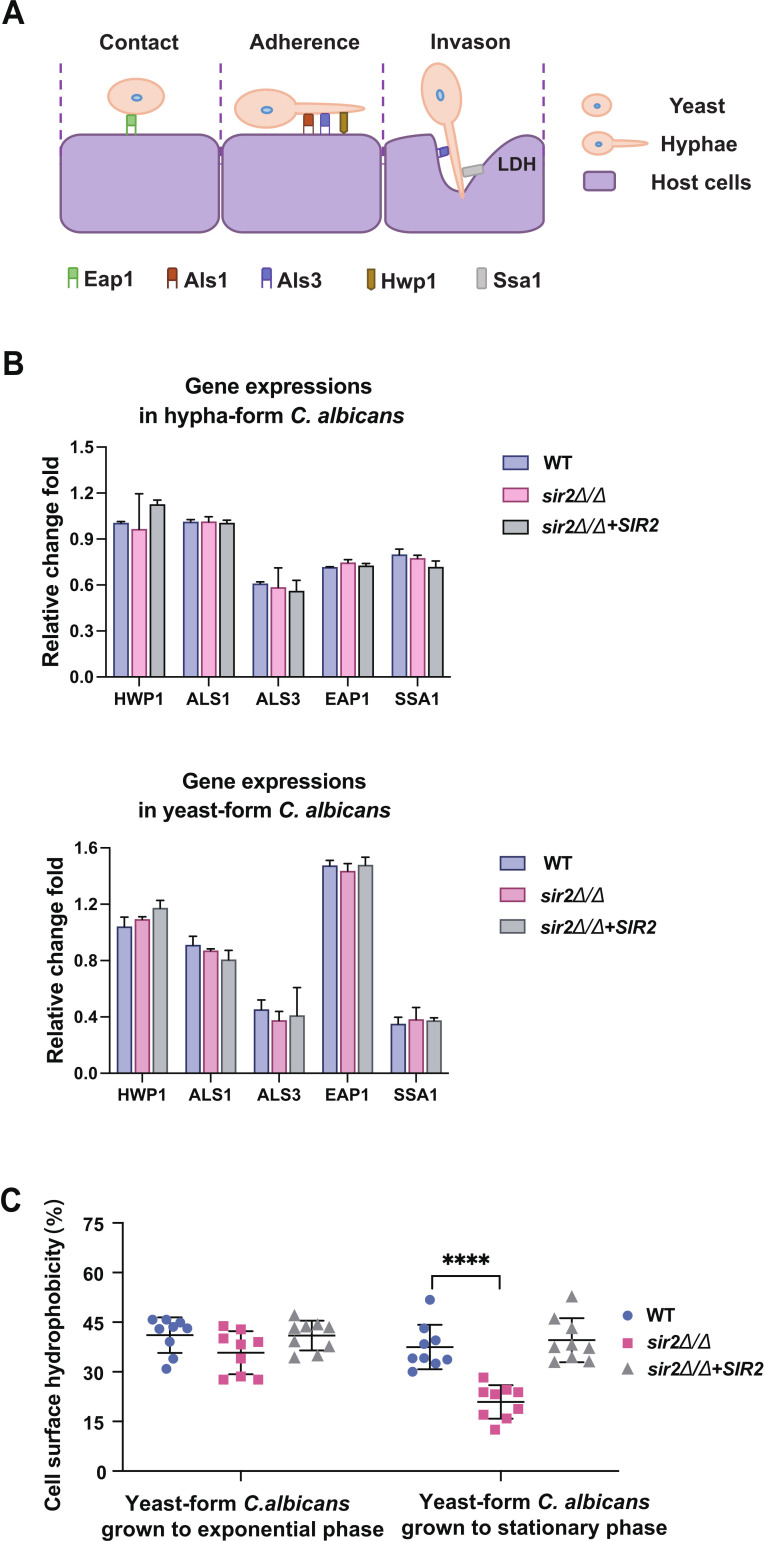
Effect of Sir2 on adhesion factors in *C. albicans* cell wall. (A) Schematic diagram of key factors involved in adhesion and invasion of *C. albicans*. (B) Differential expression of genes involved in adhesion and invasion of both hyphae- and yeast-phase *C. albicans*. Data were collected in three independent replicate experiments. The mRNA levels were normalized on the basis of *HWP1* expression levels in yeast-form or hypha-form WT, and analyzed using the one-way ANOVA (WT vs *sir2*∆*/*∆, WT vs *sir2*∆*/*∆+*SIR2*, and *sir2*∆*/*∆ vs *sir2*∆*/*∆+*SIR2*). Average values plus error bars are shown in the histogram. (C) Cell surface hydrophobicity of yeast-form *C. albicans* cultured in YPD medium at 30°C. The data represent the average of three independent replicates, with error bars. Asterisks show statistically significant differences (^****^, *P* < 0.001) based on one-way ANOVA (WT vs *sir2*∆*/*∆, WT vs *sir2*∆*/*∆+*SIR2*, and *sir2*∆*/*∆ vs *sir2*∆*/*∆+*SIR2*).

Both cell wall adhesion proteins and cell surface hydrophobicity contribute to *C. albicans* adhesion to host cells (33, 34). First, we measured the expression levels of important adhesion genes (*EAP1*, *ALS1*, *ALS3*, *HWP1*, and *SSA1*) in hyphae- and yeast-phase *C. albicans* (Fig. 3A). RT-qPCR revealed no significant differences in the expression of these genes among the tested strains (Fig. 3B). Moreover, *ALS3* and *SSA1* are key factors in the tissue internalization of *C. albicans* hyphae, which damages tissue cells and reduces LDH release. Thus, we propose that the differences in tissue damage observed in our mouse model of disseminated candidiasis were not caused by changes in the expression levels of these adhesins.

Subsequently, we determined the cell surface hydrophobicity of *C. albicans* in exponential phase and stationary phase. Our results showed that when these strains were grown to stationary phase, the cell surface hydrophobicity of *sir2*∆/∆ was lower than that of the WT and *sir2*∆/∆*+SIR2* strains, but no obvious differences were observed in these strains at exponential phase. (Fig. 3C). These results were similar to the findings in the coculture model of *C. albicans* L-929 cells, suggesting that the attenuated adhesion properties of *sir2*∆/∆ are closely related to the decrease in cell surface hydrophobicity. Previous studies demonstrated that, compared with hydrophilic cells, hydrophobic cells are not only more adherent to hosts but are also more resistant to phagocytosis and more competent at germination (35, 36). Therefore, we propose that *sir2*∆/∆ displays low virulence in a mouse model of disseminated candidiasis due to its hydrophilic surface.

### Sir2 modulates the exposures of cell wall glycans

**Fig 4:**
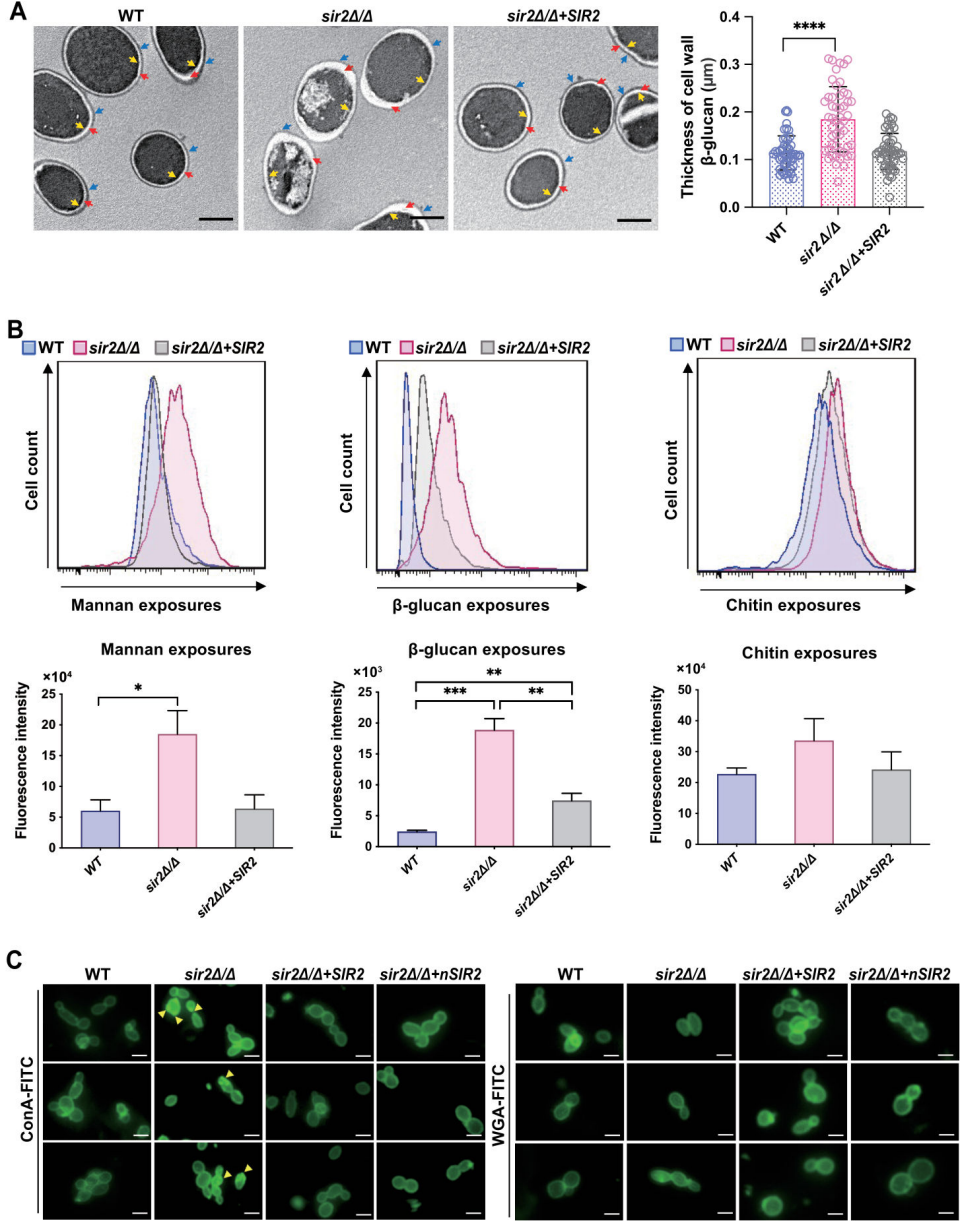
Sir2 masks the exposures of cell wall mannan and β-glucan but did not affect cell wall integrity. (A) TEM images (left) and statistical results of thickness quantification of cell wall β-glucan (right) of various yeast-phase *C. albicans* strains grown in YPD media. Mannan layer: the area indicated by blue arrows; β-glucan layer: the area indicated by red arrows; chitin layer: the area indicated by yellow arrows; bar: 200 nm. (B) Flow cytometry analysis of mannan, β-glucan, and chitin exposures in cell walls of various *C. albicans*. Histogram plots are representative of data collected in three independent replicate experiments, with error bars (above). Measurements were analyzed using the one-way ANOVA (WT vs *sir2*∆*/*∆, WT vs *sir2*∆*/*∆+*SIR2*, and *sir2*∆*/*∆ vs *sir2*∆*/*∆+*SIR2*). Asterisks show statistically significant differences (^*^, *P* < 0.05; ^**^, *P* < 0.01; ^***^, *P* < 0.001) (below). (**C**) Fluorescence staining of mannan and chitin exposures in cell walls of various yeast-phase *C. albicans*. FITC-conjugated concanavalin A (ConA-FITC, specifically binding cell wall mannan)-stained images (left); FITC-conjugated wheat germ agglutinin (WGA-FITC, specifically binding cell wall chitin)-stained images (right). Increased mannan exposures: the area indicated by yellow arrows; bar: 5 µm. Images are representative of three independent replicate experiments.

The cell surface hydrophobicity of *C. albicans* is related to the glycans in its cell wall (37). Thus, we evaluated the changes in the expression of mannan, β-glucan, and chitin, which are the main glycan components of the *C. albicans* cell wall. TEM images revealed major differences in cell wall structures among the strains (Fig. 4A). Strikingly, the thickness of the β-glucan layer in *sir2*∆/∆ was much greater than that in either WT or *sir2*∆/∆*+SIR2*. Moreover, *sir2*∆/∆ was distinguishable from the other tested strains based on the characteristics of the mannan layer, which was slightly thinner and showed less smooth morphology. However, the distributions of inner layer chitin did not significantly differ among the three strains. Additional flow cytometry analyses revealed that the exposures of mannan and β-glucan in the *sir2*∆/∆ strain were greater than those in either WT or *sir2*∆/∆*+SIR2* strains, but there were no differences in chitin exposures among the strains (Fig. 4B). Notably, compared with WT, the β-glucan exposure of *sir2*∆/∆*+SIR2* did not fully regain. Thus, we also evaluated the exposure of the *sir2*∆/∆*+nSIR2* strain to β-glucan by flow cytometry. The β-glucan exposure level of *sir2*∆/∆*+nSIR2* also returned to that of WT (Fig. S8), which further demonstrated the *SIR2* dependence on the promoter. ConA-FITC-stained and WGA-FITC-stained images also demonstrated greater exposure to mannan and no difference in exposure to chitin in *sir2*∆/∆, compared to those in WT and *sir2*∆/∆*+SIR2* (Fig. 4C). These results suggest that Sir2 affects the distribution and exposure of glycans, especially mannan and β-glucan, in the *C. albicans* cell wall. *C. albicans* hydrophobicity is closely correlated with cell surface protein mannosylation. A previous study demonstrated that a high abundance of acid-labile mannans with fibrous profiles was present on the surface of hydrophobic *C. albicans*, whereas the mannans on the surface of hydrophilic cells were mainly acid-stable mannans with shorter and more compact fibrils (33). In addition, we found that Sir2 does not affect cell wall integrity (Fig. S9). Therefore, in combination with our analysis of the cell wall, we propose that the decrease in the surface hydrophobicity of *sir2*∆/∆ is due to the increase in the abundance of acid-resistant mannans.

### Sir2 modulates host immunity

**Fig 5:**
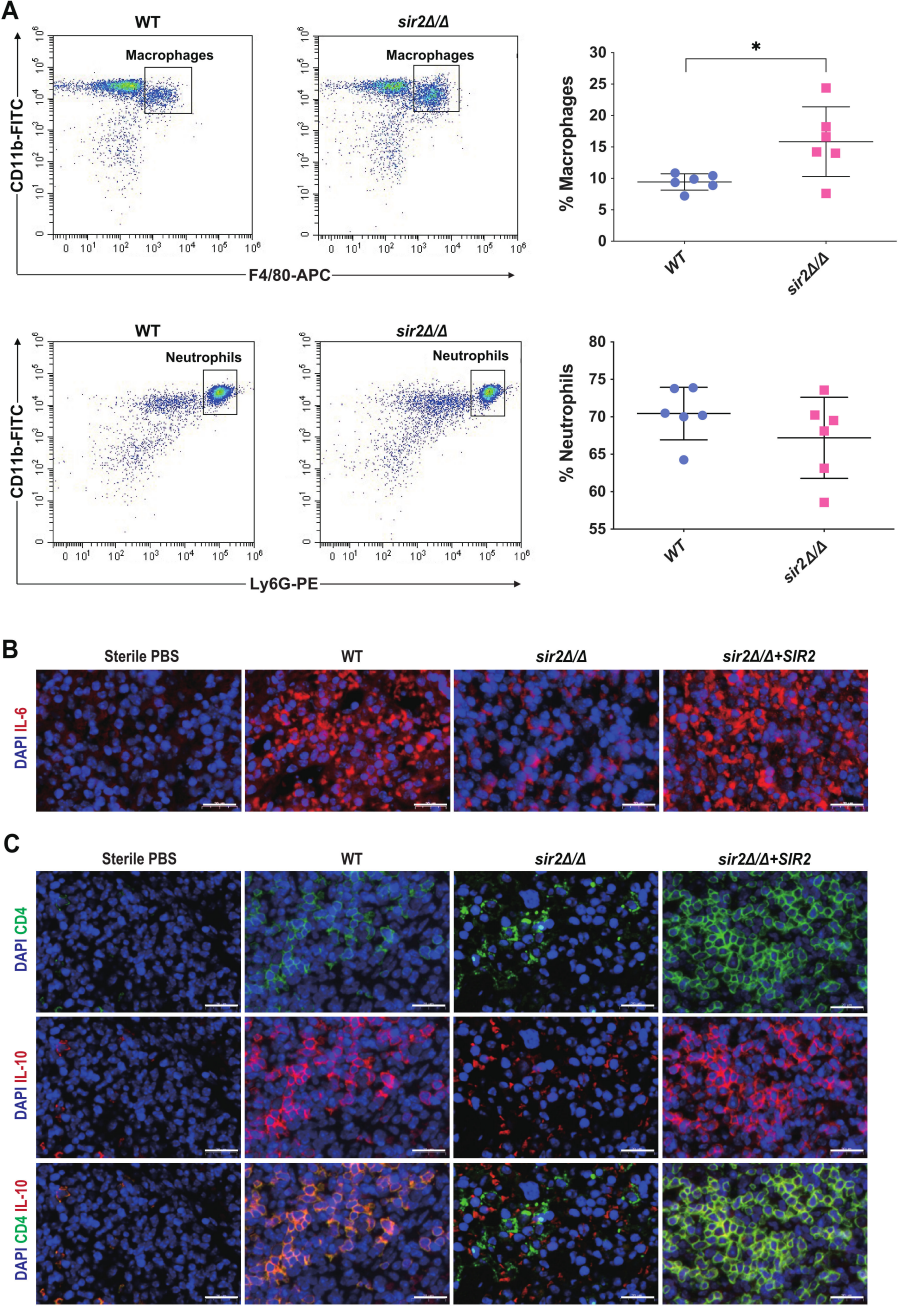
Sir2 modulates host immunity. (A) Flow cytometry assay. Percentage of macrophages and neutrophils recruited to the site of injection in BALB/c mice intraperitoneally infected with various *C. albicans* strains (for each group, *n* = 6 mice). Dotplots are representative of data collected in one of the six mice, neutrophils: CD11b^+^, F4/80^−^, and Ly-6G^+^), macrophages: CD11b^+^, F4/80^+^, and Ly-6G^−^ (left). Measurements were analyzed using the one-way ANOVA and average values plus error bars are shown in scatter diagram. Asterisks show statistically significant differences (^*^, *P* < 0.05) (right). (B) Fluorescence micrographs of interleukin-6 (IL-6) secreted from mice spleen after 2 days of infection by various *C. albicans* strains; DAPI: staining DNA to localization cells, rat anti-IL-6 antibody to immune IL-6; bar: 20 µm. (C) Fluorescence micrographs of T helper type 2 (Th2) cells from mice spleen after 2 days of infection by various *C. albicans* strains; DAPI: staining DNA to localization cells, Th2: CD4/IL-10; bar: 20 µm.

During infection with *C. albicans*, mannan and β-glucan, which act as PAMPs in the cell wall, activate and modulate the initial response of the innate immune system. Thus, we explored the differences in immune cell recruitment between *sir2*∆/∆ and WT mice after intraperitoneal injection into the peritoneal cavity. Flow cytometry analysis revealed that *sir2*∆/∆ mice recruited significantly more macrophages (CD45^+^, CD11b^+^, and F4/80^+^) than did WT mice. However, no differences were observed in the number of neutrophils (CD45^+^, CD11b^+^, and Ly6G^+^) collected between the groups (Fig. 5A). These results confirmed that Sir2 facilitates *C. albicans* escape from macrophages.

Furthermore, a hyperinflammatory state, which induces collateral host injury at the tissue and organ level, has been observed in patients with invasive *C. albicans* infections, leading to adverse outcomes (38). In fact, hyperinflammation may be a consequence of an unnecessarily prolonged or exaggerated proinflammatory immune responses post-infection (39). Here, we investigated the secretion of the inflammatory factor interleukin-6 (IL-6) in lymphoid organs in a mouse model of disseminated candidiasis. The results showed that *sir2*∆/∆ stimulated much less production of IL-6 than did WT and *sir2*∆/∆*+SIR2* (Fig. 5B).

Based on Sir2 regulation of macrophage recognition, we hypothesized that deletion of Sir2 accelerates the clearance of immune cells against *C. albicans*, thereby preventing hyperinflammation in mouse tissues. In addition, severe systemic stress and overwhelming microbial inoculation cause the immune system to mount a T helper type 2 (Th2) lymphocyte response that is accompanied by the secretion of IL-10 in an infection normally controlled by Th1 immunity, resulting in immunosuppression (40). Here, immunofluorescence was performed to investigate Th2 cells (CD4/IL-10) in lymphoid organs after *C. albicans* infection. The number of Th2 cells in the spleens of mice treated with *sir2*∆/∆ was much lower than that in the spleens of mice treated with WT or *sir2*∆/∆*+SIR2* and was comparable to that in the PBS control group (Fig. 5C). Overall, we believe that Sir2 participates not only in *C. albicans* immune escape but also in the colonization and invasion of *C. albicans*, in which Sir2 can further trigger host immune tolerance.

### Sir2 involves in alternative carbon utilization under hypoxia environment

**Fig 6:**
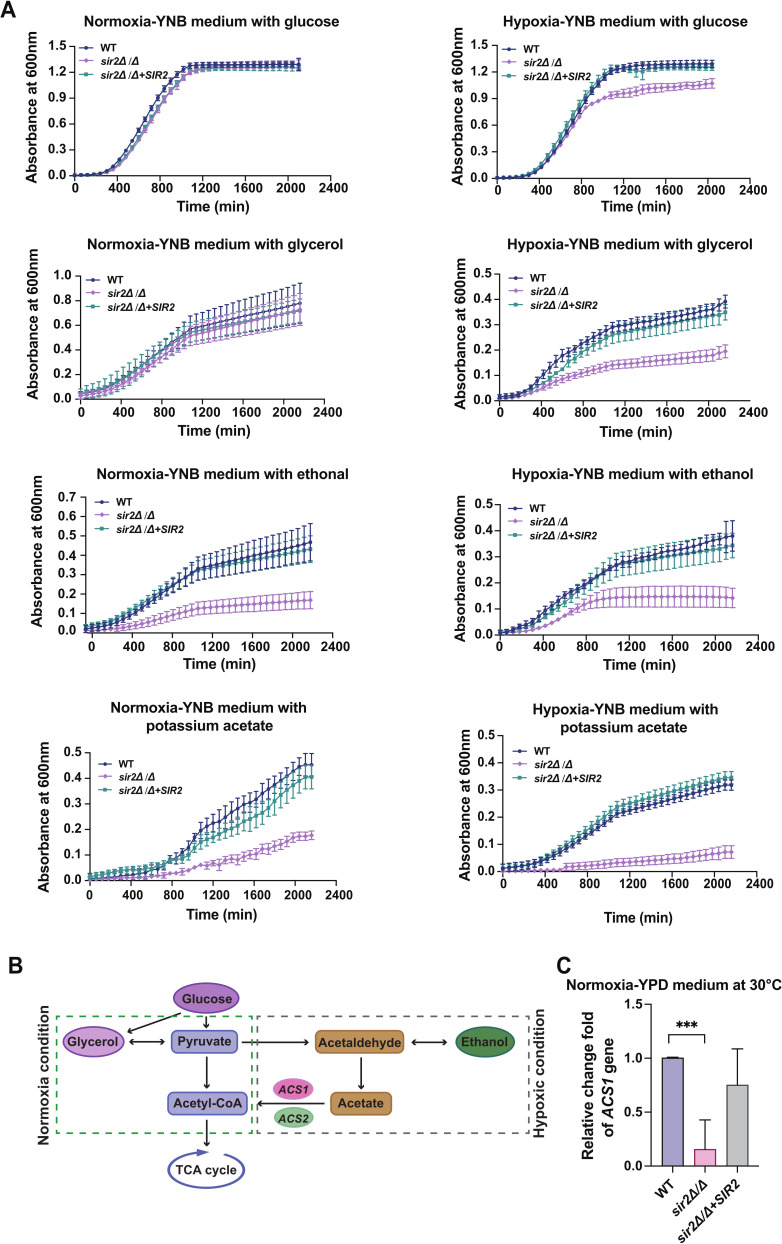
Sir2 involves in the regulatory of alternative carbon utilization. (A) Growth curves of different strains in YNB medium containing 2% glucose (mass/vol), 2% glycerol (vol/vol), 2% ethanol (vol/vol), or 2% potassium acetate (vol/vol) and incubated at 30°C under normoxia or hypoxic condition for 36 h. The initial inoculation was an absorbance of 0.05 at 600 nm. Results are representative of three independent replicate experiments. (B) Schematic view of the respiratory path (metabolism under normoxia condition) and pyruvate bypass route (metabolism under hypoxic condition). (C) Differential expression of *ACS1* in yeast-form *C. albicans* cultured in YPD medium at 30°C under normoxia, which involves the glycolysis pathway by regulating alternative carbon utilization under hypoxic condition. Data were collected in three independent replicate experiments and analyzed using the one-way ANOVA (WT vs *sir2*∆*/*∆, WT vs *sir2*∆*/*∆+*SIR2*, and *sir2*∆*/*∆ vs *sir2*∆*/*∆+*SIR2*). Asterisks show statistically significant differences (^***^, *P* < 0.001). Average values plus error bars are shown in the histogram.

Sir2 has been shown to interact with Hxk1, which is involved in the regulation of GlcNAc metabolism (18). Thus, we hypothesized that Sir2 is involved in *C. albicans* metabolism. To verify this hypothesis, *C. albicans* growth in media supplemented with various carbon sources was examined under hypoxic and normoxic conditions. A spot dilution assay showed that the carbon utilization capacity of *sir2*∆/∆ was slightly poorer than that of *sir2*∆/∆*+SIR2* and WT under hypoxic conditions; however, there were no differences among the three strains under normoxic conditions (Fig. S10). To further demonstrate this result, *C. albicans* metabolism in liquid media supplemented with different carbon sources was evaluated *via* growth curves. Under hypoxic conditions, *sir2*∆/∆ exhibited significant growth restriction, while under normal oxygen conditions, its growth trend was similar to that of control strains, apart from in ethanol and potassium acetate (Fig. 6A). Based on these results and information about the critical glycolysis processes (Fig. 6B), we measured the expression of the *ACS1* gene of yeast-form *C. albicans* cultured in YPD medium at 30°C (under normoxic condition), which encodes the acetyl coenzyme A (acetyl-CoA) synthetase Acs1, and is a key factor in alternative carbon utilization under hypoxic conditions (41, 42). RT-qPCR revealed that the expression of *ACS1* was significantly lower in *sir2*∆/∆ than in WT and *sir2*∆/∆*+SIR2* (Fig. 6C). In fact, *C. albicans* must be able to assimilate to alternative carbon sources in its environment to generate sufficient energy and metabolites to survive. Therefore, we propose that the survival and colonization of *C. albicans* strains lacking SIR2 are reduced at the site of hypoxic infection.

## DISCUSSION

In this study, we demonstrated that Sir2 was required for normal infection levels during hematogenously disseminated candidiasis. Furthermore, in a mouse model of disseminated candidiasis, we found *C. albicans* hyphae only in kidneys of mice infected with either the *SIR2* complemented strain or the WT strain (Fig. 1E ; Fig. S4D). In this study, Sir2 had no significant effect on hyphal development under hyphal-inducing conditions (Fig. 2A; Fig. S6). Although Sir2 was reported in two previous studies to be involved in regulating the morphology transition of *C. albicans*, the hyphal-inducing conditions and strains previously reported differ from those we used (17, 22). Furthermore, Sir2 has been reported to regulate genome stability by repressing recombination at the rDNA locus, and this regulatory mechanism is plastic because different environmental stress conditions lead to general genome instability and mask Sir2-mediated recombination control (20). This mechanism explains the different regulatory effects of Sir2 on hyphal development under different hyphal-inducing conditions.

In addition to hyphal formation, the ability of yeast cells to adhere to host cells is also important for *C. albicans* invasion of tissues. An *in vitro* flow adhesion assay showed that yeast forms bound to confluent host cells in significantly greater numbers than pseudohyphal and hyphal forms under flow conditions (43). Another previous study reported that *C. albicans* lacking hyphae was capable of establishing restricted zones of infection and colonization instead of being cleared by the immune system in mice (44). In our study, we found that deletion of *SIR2* significantly reduced the capacity of *C. albicans* to adhere to host cells (Fig. 2B and C). Thus, we suggest that the loss of adhesion in *sir2*∆/∆ is an important reason why it rarely colonizes mouse models of disseminated candidiasis. Furthermore, we demonstrated that the differences in adhesion among the strains were consistent with changes in cell surface hydrophobicity (Fig. 3C). We also found that deletion of *SIR2* increased the exposure of mannan, which is an important determinant of cell surface hydrophobicity (Fig. 4B and C). In addition, the outermost morphology of *sir2*∆/∆ indicates that it is an acid-stable hydrophilic mannan (Fig. 4A). Therefore, we assumed that the reduced adhesion of *sir2*∆/∆ is the result of its decreased cell surface hydrophobicity caused by increased exposure of acid-stable mannan on the cell wall.

During infection, the cell wall glycans of *C. albicans* not only affect adhesion but are also important fungal PAMPs that activate host innate immune responses (25, 45). Our findings from the phagocyte recruitment assay demonstrated that *sir2*∆/∆ stimulated an enhanced immune response in macrophages in BALB/c mice (Fig. 5A), which was consistent with the changes in cell wall glycans. In addition, a previous study revealed that virulence-deficient *C. albicans* was more easily cleared by the host immune system and less susceptible to immune tolerance (39), which is also consistent with our findings from tissue immunofluorescence. We found that *sir2*∆/∆ induced a weaker inflammatory response and less immune tolerance in the mouse spleen than did WT and *sir2*∆/∆+*SIR2* (Fig. 5B and C). Based on these results, we propose that deletion of *SIR2* causes changes in cell wall glycans in *C. albicans*, thereby enhancing innate immunity and reducing susceptibility to immune tolerance, which promotes rapid immune clearance.

A previous study reported that differential chromatin states mediated by sirtuins control gene expression and alter the cell’s metabolic state upon environmental changes (21, 22). Our results showed that Sir2, a sirtuin, regulated the expression of the metabolic gene *ACS1* and was required for the normal growth of *C. albicans* under hypoxic conditions (Fig. 6), suggesting that the Sir2-mediated chromatin state is linked to adaptation to a hypoxic environment. Tissues often experience hypoxia at the site of infection or injury (46). Therefore, we suggest that the attenuated virulence of *sir2*∆/∆ might be correlated with the role of Sir2 in *C. albicans* metabolism under hypoxic conditions, in addition to its lack of adhesion and immune escape abilities. In fact, HDACs, as histone-modifying enzymes, can both directly and indirectly affect genome function (47). The direct effect on gene expression occurs via the alteration of chromatin structure, while the indirect influence on gene transcription occurs through the binding of gene products to effector proteins or chromatin remodeling complexes. Since Sir2 is dispensable for repressing recombination at the rDNA locus (20), we suggest that Sir2 indirectly regulates the expression of the ACS1 gene to promote *C. albicans* survival in a hypoxic environment.

Interestingly, although Sir2 interacts directly with hxk1 (18), *sir2*∆/∆ and *hxk1*∆/∆ differ in cell wall characteristics. *sir2*∆/∆ cells displayed a change in cell wall mannan and β-glucan exposure, while *hxk1*∆/∆ showed altered cell wall chitin synthesis. Additionally, Hxk1 has been shown to perform several functions, such as inhibiting the yeast-hyphal transition and promoting metabolic gene expression. In our study, although Sir2 also plays a regulatory role in *C. albicans* metabolism, *sir2*∆/∆ exhibited growth defects under hypoxic conditions, while *hxk1*∆/∆ disrupted the GlcNAc catabolic pathway compared with that of WT. Our results and the available literature suggest that the effect of Sir2 on *C. albicans* virulence in a mouse model of disseminated candidiasis is not related to Hxk1 regulation of the morphology and metabolism of *C. albicans*.

In conclusion, our work demonstrated that Sir2 is required for *C. albicans* virulence, and we propose that Sir2-mediated *C. albicans* virulence is associated with many factors, including adhesion and host immune escape mediated by the cell wall of *C. albicans* itself as well as metabolic activity under hypoxic conditions. These findings emphasize the importance of Sir2 in the development of antifungal drugs and the development of new therapeutic strategies for candidiasis.

## MATERIALS AND METHODS

### Plasmid and strain construction

The oligonucleotides and the primers for constructing and detecting plasmids are given in Table S1. Wild-type *C. albicans SC5314* was used to generate Sir2 null mutant (*sir2*∆*/*∆). The pV1093 plasmid (Addgene plasmid #111428), a gift from Gerald Fink, was used to construct a *C. albicans* CRISPR system targeting the *SIR2* gene according to the method described previously to create *sir2*∆*/*∆ (Fig. S1) (48); details are presented in the supplementarl material, and disruption of *SIR2* was confirmed by PCR and sequencing. To generate reintegrant control strains expressing *SIR2* under MET3 promoter (*sir2*∆*/*∆*+SIR2*) or *SIR2*’s own promoter (*sir2*∆*/*∆*+nSIR2*), *SIR2* ORF (the guide sequence of *SIR2* was replaced by their synonymous codons) and *SIR2* ORF plus promoter regions (contained 2,000 bp regions upstream of *SIR2* ORF) were cloned into modified pCaEXP plasmid containing *RP10* locus, *MET3* promoter and zeocin resistance sequence (49) and modified pCaEXP plasmid without *MET3* promoter, respectively (Fig. S2). These constructions were transformed into *sir2*∆*/*∆. The integration of constructions into the targeted loci was confirmed by PCR and RT-qPCR.

### Mouse models of hematogenously disseminated candidiasis and pathogenicity evaluation

The virulence of the various strains was tested in the mouse model of hematogenously disseminated candidiasis as described previously (50). BALB/c female mice (*n* = 10 per group, 8–12 weeks old, animal certification number: XJTUAE2019-544) were injected via the lateral tail vein with 1 × 10^6^ yeast-phase cells of various *C. albicans* resuspended in 100 µL of sterile PBS and 100 µL of sterile PBS as the control. The fungal inocula were randomly allocated to groups. These mice were monitored at least two times daily and moribund mice were euthanized. To determine the organ damage, 10 mice were inoculated with each strain as in the survival experiments. After 2 days of infection, two mice were randomly selected from each group for the histopathological examination of the livers and kidneys by H&E staining and PAS staining. Tissues used for histopathological examination were embedded in wax. To determine organ fungal burden, the brain, spleen, liver, and kidney of remaining mice from each group were harvested, weighed, separately homogenized with a tissue grinder, and quantitatively cultured by incubating serial dilutions on YPD agar plates.

### Hypha growth

Yeast-phase *C. albicans* grown overnight in YPD medium were adjusted to a final density of 1 × 10^5^ cells/mL in RPMI 1640 medium with 10% FBS. Cultures were incubated at 37°C. At 0, 2, 5, and 24 h, aliquots were transferred to glass slides and stained with calcofluor white (CFW, Sigma-Aldrich). Images were captured using fluorescence microscope (ZEISS), and hyphal lengths and frequencies shown in representative areas were counted using ImageJ.

### Adhesion on host cells

To analyze the adhesions of various strains to L-929 cells, the coincubation model of host cells-*C. albicans* was established as described in the previous study (51). Briefly, L-929 cells labeled by DiI (10 µM) grew to 80–90% confluency and then inoculated with 1 × 10^5^ yeast-phase of *C. albicans* stained by CFW (10 µM). The infected cells were incubated in 5% CO_2_ at 37℃. After a certain time of incubation (0.5, 1.5, and 2 h), medium was discarded and non-adherent organisms were removed. The cells were fixed with 4% paraformaldehyde and viewed under fluorescence microscope (ZEISS). The quantification of attached *C. albicans* was performed by the measuring fluorescence intensity of each well using Cytation imaging reader (BioTek). The wells just containing L-929 cells were as control. All the controls and experimental samples were set in quintuplicate wells and three independent experiments were conducted.

### Host cell damage assay

To evaluate the damages of L-929 cells caused by *C. albicans*, the releases of LDH were determined after 24 h of co-incubation of host cells-*C. albicans* using LDH cytotoxicity assay kit (Thermo Scientific) according to the manufacturer’s recommendations, and details presented in Supplementary Materials. The co-incubation model was established as described in theMaterials and Methods section. All the control and experimental samples were set in triplicate wells and three independent experiments were conducted.

### Gene expression analysis

*C. albicans* were grown in YPD medium at 30°C or RPMI 1640 medium with 10% FBS at 37°C until they reached the exponential phase, and then the gene expression levels were determined by RT-qPCR. The results were analyzed by the 2^−△△CT^ method (52), 18S rRNA as the endogenous control. The primers are given in Table S2. All the gene expression levels were determined in three biological replicates, each tested in triplicate.

### Cell surface hydrophobicity

The cell surface hydrophobicity was measured by a modified microbial adhesion to hydrocarbon test (53). Briefly, *C. albicans* were grown in YPD medium until they reached the exponential or stationary phases at 30°C. Then yeast-phase cells were harvested and prepared a suspension in PBS with an optical density of 0.4 at 492 nm. Thereafter, 1 mL of xylene was added to each 3 mL of suspension. The phases were mixed by vortexing for 1 min and left for 5 min until the two phases separated. The relative cell surface hydrophobicity was expressed as the percentage reduction of initial turbidity of the aqueous suspension. All assays were made in three biological replicates.

### TEM

Yeast-phase *C. albicans* grown overnight in YPD medium were pelleted and sequentially fixed. Samples were dehydrated using increasing concentrations of ethanol and subsequently embedded in epon. Afterward, ultrathin sections were prepared and cell walls were observed by TEM (Hitachi H-7650, Japan) and the thickness of β-glucan shown in the representative area was counted using ImageJ.

### Cell wall immunolabeling

The exposures of β-glucan, mannan, and chitin in yeast-phase *C. albicans* were analyzed (54). Briefly, for β-glucan, the CLEC7A polyclonal antibody (primary antibody, 1:100, ABclonal) was added to *C. albicans* and incubated at 4°C overnight. Then, the FITC-conjugated goat anti-rabbit IgG (H+L) (secondary antibody, 1:100, ABclonal) was added after washing, and then incubated for 1 h in the dark at room temperature. For mannan or chitin, staining was performed with 200 µg/mL of FITC-conjugated concanavalin A (ConA-FITC) or FITC-conjugated wheat germ agglutinin (WGA-FITC) (Sigma-Aldrich), specifically binding-mannan or chitin on yeast cell walls. Ten thousand events were collected and examined by flow cytometry (Beckman Coulter, CytoFLEX) and fluorescence microscope. All assays were made in three biological replicates.

### Spot dilution assay

Yeast-phase *C. albicans* grown overnight in YPD medium were adjusted to 5 × 10^7^ cells/mL with sterile ddH_2_O, and 10-fold serially diluted and then 5 µL of each sample was spotted onto YPD or YNB agar plates containing different agents (the details of agents are shown in the supplemental material). The plates were incubated at 30°C for 2–6 days and photographed.

### Neutrophil and macrophage recruitment assay

The ability of *C. albicans* to recruit neutrophils and macrophages was performed by the method reported previously (55). BALB/c female mice (*n* = 6 per group, 8–12 weeks old) were randomly assigned to groups. Yeast-phase *C. albicans* overnight in YPD medium were resuspended in 100 µL sterile PBS (1 × 10^7^ cells) and then injected intraperitoneally. After 4 h, mice were euthanized. Immune infiltrates in the peritoneal cavity were collected by peritoneal lavage. Cells were labeled to distinguish neutrophils and macrophages using corresponding antibodies (Alexa Fluor 700 anti-mouse CD45, FITC anti-mouse CD11b, PE anti-mouse Ly-6G, and APC anti-mouse F4/80, BioLegend) according to the manufacturer’s recommendations. 10,000 cells were analyzed for each mouse using flow cytometry. Data represent percent immune cells, neutrophils (CD45^+^, CD11b^+^, F4/80^−^, and Ly-6G^+^) and macrophages (CD45^+^, CD11b^+^, F4/80^+^, and Ly-6G^−^).

### Tissue immunofluorescence

The spleens from mouse models of disseminated candidiasis were separated after infection for 2 days. Tissues embedded in wax were used to the examination of macrophage recruitment and the release of cytokines. Formalin-fixed paraffin-embedded spleen sections (4 μm thick) were stained following standard protocols; detailed descriptions are given in the supplemental material. Sections were analyzed by fluorescent microscopy.

### Growth curves

Yeast-phase *C. albicans* overnight in YPD medium were harvested and washed by PBS, and adjusted to an absorbance of 0.05 at 600 nm in YNB medium with 2% glucose, glycerol, ethanol, or acetate. Growth was assessed in a 96-well plate using a plate reader at 30°C under normoxic or hypoxic conditions, with continuous shaking and readings at 600 nm taken every 60 min for 36 h. All assays were made in three biological replicates.

### Statistical analysis

All of the data were plotted and analyzed for statistical significance using GraphPad Prism v.9.3.1. The data were compared using a one-way analysis of variance (ANOVA) and multiple unpaired *t* test, depending on whether the data were normally distributed. The graphs are annotated to indicate the levels of the statistical significance of the results (^*^P < 0.05, ^**^P < 0.01, ^***^P < 0.001, and ^****^P < 0.0001).

## Data Availability

Main study data are included in the article and/or supplemental material. Further information and requests for resources and reagents should be directed to Peipei Zhang.

## SUPPLEMENTAL MATERIAL

Supplemental material mbio.00445-24-s0001.docx Fig. S1-S10; Tables S1-S2.

ASM does not own the copyrights to Supplemental Material that may be linked to, or accessed through, an article. The authors have granted ASM a non-exclusive, world-wide license to publish the Supplemental Material files. Please contact the corresponding author directly for reuse.
