# Supplementary material for: Histone deacetylase Sir2 promotes the systemic Candida albicans infection by facilitating its immune escape via remodeling the cell wall and maintaining the metabolic activity
Source: mBio. 2024 Apr 29;15(6):e00445-24. doi: 10.1128/mbio.00445-24 (PMC11237532; doi:10.1128/mbio.00445-24)
Supplement: Supplemental material — Fig. S1-S10; Tables S1-S2. [file mbio.00445-24-s0001.docx]

**Histone deacetylase Sir2 promotes the systemic *Candida albicans* infection by facilitating its immune escape via remodeling the cell wall and maintaining the metabolic activity**

**1. Supplementary Materials and Methods………………………………2**

1.1 Plasmid and strain construction………………………………………………2

1.2 Host cell damage assay……………………………………………………………3

1.3 Spot dilution assay…………………………………………………………………3

1.4 Tissue immunofluorescence………………………………………………………4

**2. Supplementary Tables and Figures……………………………………5**

2.1 Oligonucleotides used to construct plasmids ……………………………………5

2.2 Primers to analysis gene expression levels………………………………………7

2.3 Schematic diagram of *SIR2* mutant strain construction………………9

2.4 Schematic diagram of *SIR2* expression plasmid construction…………………11

2.5 PCR verification and *SIR2* gene expression levels………………………………13

2.6 *In vivo* virulence of complemented strain expressing *SIR2* under its own promoter15

2.7 Hyphae frequencies………………………………………………………………17

2.8 Hyphae formation and development……………………………………………18

2.9 Adhesion on host cells……………………………………………………………20

2.10 Exposure of cell wall β-glucan…………………………………………………21

2.11 Cell wall integrity………………………………………………………………22

2.12 Alternative carbon utilization analyzed using spot dilution assay………………23

**3. References………………………………………………………………24**

**1. Supplementary Materials and Methods**

**1.1 Plasmid and strain construction**

CRISPR system was used to construct *SIR2* deletion strain (*sir2*$\Delta$*/*$\Delta)$. In details, two complementary oligos (Table S1) matched the SIR2 gene with restriction sites were synthesized by Beijing Tsingke Biotech Co., Ltd. To obtain guide sequence of *SIR2*, the oligos were annealed at 70°C for 20 min. Then the guide sequence was ligated to the pV1093 plasmid(*1*) ﻿digested with *BsmB*I (New England Biolabs, NEB) by T4 ligase (New England Biolabs, NEB) at 37°C. Correct guide expression plasmids (pV1093-SIR2) were confirmed by sequencing. The guide expression plasmids were linearized by digesting ﻿with *Kpn1* and *Sac1* (New England Biolabs, NEB) before transformation for efficient targeting to the *ENO1* locus. The repair templates were amplified with 485 bp upstream and 502 bp downstream of the open reading frame (ORF) of *SIR2* gene using primers (Fig. S1 and Table S1). The PCR products of upstream and downstream were ligated by overlap PCR and purified by 1% agarose gel. To create *sir2*$\Delta$*/*$\Delta$, the linearized pV1093-SIR2 plasmids (10 μg) were transformed into WT by electrotransformation and were ﻿selected using YPD plates with nourseothricin at a concentration of 200 μg/ml. The genomes of positive colonies were extracted after growing in YPD medium overnight and CRISPR-mutagenized loci were verified by sequence analysis of PCR products amplified from the target locus. To create the *sir2*$\Delta$*/*$\Delta$*+SIR2* and *sir2*$\Delta$*/*$\Delta$*+nSIR2* stains, the constructed modified pCaEXP plasmids containing SIR2 ORF or SIR2 ORF containing own promoter were transformed into *sir2*$\Delta$*/*$\Delta$ and screened on the YPD containing geneticin (200 μg/ml).

**1.2 Host cell damage assay**

The releases of enzyme lactate dehydrogenase (LDH) from L-929 cells after 24 h of co-incubation of host cells and *C. albicans* were detected using pierce LDH cytotoxicity assay kit (Thermo Scientific). Initially, the host cells were seeded in 96-well plates with an overnight culture. Next, 100 μl of MEM medium (as host cells spontaneous control) and 100 μl of the *C. albicans* (10^6^ cells/ml, WT, *sir2*$\Delta$*/*$\Delta$ and *sir2*$\Delta$*/*$\Delta$*+SIR2*) were added to the 96-well plate containing host cells. To correct for spontaneous release of LDH from *C. albicans* cells, the same as numbers of *C. albicans* cells used in the experimental wells were add to clear wells (no host cells) and adjust the final volume to 100 μl/well with MEM medium. After that, the 96-well plate was incubated at 37℃, 5% CO_2_ for 24 h. Forty-five minutes before harvesting the supernatant, 10 μl of lysis buffer (10×) was add to wells containing host cells as the maximal LDH release control and10 μl of sterile PBS was add to other wells to correct the volume. 50 µl of each sample medium was transformed to a new 96-well plate containing 50 µl of reaction mixture and then they were incubated at room temperature for 30 min protected from light. To determine LDH activity, 50 µl of stop solution was add to each sample well and mix by gentle tapping, the absorbance value was got by subtracting the 680 nm absorbance value (background) from the 490 nm absorbance before calculation of % cytotoxicity according to the formula shown as follow:

$\%Cytotoxicity=\frac{Experimental value-C. albicans spontaneous contol-host cells spontaneous control}{host cells maximum control-host cells spontaneous control}$×100

**1.3 Spot dilution assay**

The preparation details of YPD agar plates and YNB agar plates were shown as follow:

YPD agar plates with or without calcofluor white (50 μM), SDS (0.002%), NaCl (0.5 M) and H_2_O_2_ (10 μM). The plates were incubated at 30℃ for 2 days and photographed.

YNB medium containing 2% glucose overnight and washed with sterile ddH_2_O. *C. albicans* were adjusted to 1×10^5^ cells and 10-fold serially diluted. Then, 2.5 µl of each sample was spotted onto YNB agar plates with 2% glucose, glycerol, ethanol or acetate. The plates were incubated at 30℃ for 4-6 days and photographed.

**1.4 Tissue immunofluorescence**

Four-μm-thick formalin-fixed paraffin embedded spleen sections were stained following standard protocols. Briefly, sections were boiled in 10 mM sodium citrate buffer (pH 6.0) in a microwave. Slides were blocked in buffer containing 0.5% Tween 20 and 0.2% goat serum. For staining, slides were boiled in retrievagen A (BD) and blocked with buffer without Tween. Next, slides were incubated with antibody overnight at 4℃. The following antibodies were used: rat anti-IL-6 antibody (1:100, Abcam) rat anti-CD4 antibody (1:100, BD Biosciences), rat anti-IL-2 antibody (1:100, Abcam) and rat anti-IL-10 antibody. Slides were incubated with the following secondary antibodies: Alexa Fluor 647 goat anti-mouse IgG (Invitrogen) or goat anti-rat horseradish peroxidase (HRP; Cambridge Bioscience) at room temperature for 1 h. Sections were counterstained with 4,6-diamidino-2-phenylindole (DAPI, Roche).

**2. Supplementary Tables and Figures**

**2.1 Oligonucleotides used to construct plasmids**

**Table S1.** Sequences and descriptions of the synthetic oligonucleotides used in this study to construction *SIR2* deletion strain (*sir2*$\Delta$*/*$\Delta)$and *SIR2* compensation strains (*sir2*$\Delta$*/*$\Delta$*+SIR2* and *sir2*$\Delta$*/*$\Delta$*+nSIR2*)

| **Primer / Oligonucleotides** | **Sequence (5’-3’)** | **Description** |
| --- | --- | --- |
| SIR2_sgRNA top | ATTTGACAATCAATCGCCAAAATGGG | Oligomers to obtain guide sequence of *SIR2* |
| SIR2_sgRNA bottom | AAAACCCATTTTGGCGATTGATTGTC |  |
| SIR2 upstream donor_F | GAACCATGATCAGATGAATGCCG | Primers to obtain repaired template (up) |
| SIR2 upstream donor_R | CATCAACTTCAACAAGATATACGGACTCGTGGTTATATTTTCTCCAGAGATG |  |
| SIR2 downstream donor_F | GGAGAAAATATAACCACGAGTCCGTATATCTTGTTGAAGTTGATGTTAACTGATTC | Primers to obtain repaired template (down) |
| SIR2 downstream donor_R | CATCTGGTGGTAAGACTAAATGAGC |  |
| SIR2 upstream donor+65_F | CAAGAATACTAGTGGTAGTGGGTC | Primers to check *SIR2* mutation |
| SIR2 downstream donor+113_R | CAGTTTCAATCCTGCTCGTTGTTC |  |
| SIR2 upstream donor-485_F | CTCTGGAGAAAATATAACCACGAGTC | Primers to check *SIR2* mutation |
| Linearized pCaEXP for Geneticin^R^_F | GGCGTAATCATGGTCATAGC | Primers to create linearized pCaEXP vector for ligation Geneticin^R^ |
| Linearized pCaEXP for Geneticin^R^_R | ATCAATGTTTAAAAGAATGGTTGAAATC |  |
| Geneticin^R^-pCaEXP_F | CTTTTAAACATTGATGTTTCCCGTTGAATATGGCTCAT | Primers to create Geneticin^R^ insert for ligation with linearized pCaEXP vector |
| Geneticin^R^-pCaEXP_R | GACCATGATTACGCCTTAGAAAAACTCATCGAGCATCAAATG |  |
| Linearized pCaEXP for SIR2_F | GATCCTCTAGAGTCGACCTGC | Primers to create linearized pCaEXP vector for ligation *SIR2* |
| Linearized pCaEXP for SIR2_R | CGGGGAGGGTATTTACTTTTAAATATAG |  |
| SIR2-ORF-pCaEXP_F | TAAATACCCTCCCCGATGACAACTTTTTGGTCACAAACC | Primers to create *SIR2* insert for ligation with linearized pCaEXP vector |
| SIR2-ORF-pCaEXP_R | CGACTCTAGAGGATCTCATTTTTTTGTCGAAGTTGATTTCTTG |  |
| SIR2-promoter-pCaEXP_F | GTGTTCATTCCCTTGAACCAAATAATGCCAAGGCAATG | Primers to create *SIR2* and promoter insert for ligation with linearized pCaEXP vector |
| SIR2-promoter-pCaEXP_R | CCAAAAAGTTGTCATTTACTAGAGGATTTCTCTCAAATAACTGAAACG |  |
| Linearized pCaEXP_SIR2_F | ATGACAACTTTTTGGTCACAAACAATC | Primers to create linearized pCaEXP vector for ligation *SIR2* plus promoter |
| Linearized pCaEXP_SIR2_R | CAAGGGAATGAACACAGTTGTGC |  |

**2.2 Primers to analysis gene expression** **levels**

**Table S2.** Sequences and descriptions of the synthetic oligonucleotides used in this study to detect the relative expression of genes involved in *SIR2*, adhesion, hyphae development, invasion host and cell wall synthesis related genes by qPCR.

| **Primers** | **Primer sequences (5'-3')** | **Descriptions** |
| --- | --- | --- |
| 18S_F | AATTACCCAATCCCGACAC | Primers of reference gene to calibrate target genes expression in *C. albicans* |
| 18S_R | TGCAACAACTTTAATATACGC |  |
| SIR2_F | TGGACCTGCAACTGGAACTG | Primers to detect the *SIR2* gene expression in WT, *sir2∆/∆* and *sir2∆/∆*+*SIR2* |
| SIR2_R | ACCTGCACCAGTAACTACCA |  |
| ALS1_F | ATTGGTAAAGTAACTGTACCA | Primers to detect the *ALS1* gene expression involved in adhesion and invasion |
| ALS1_R | CACATTGAATATGCCATGTG |  |
| ALS3_F | CCTGAAATTGACATGTAGCA | Primers to detect the *ALS3* gene expression involved in adhesion and invasion |
| ALS3_R | CTAATGCTGCTACGTATAATT |  |
| HWP1_F | TGGTGCTATTACTATTCCGG | Primers to detect the *HWP1* gene expression involved in adhesion and invasion |
| HWP1_R | CAATAATAGCAGCACCGAAG |  |
| ECE1_F | GCCATCATCCACCATGCTCCAG | Primers to detect the *ECE1* gene expression involved in biofilm formation, damage host epithelial membranes and activation epithelial immunity |
| ECE1_R | CAGGAACAGTAGGTGCTTGGTCAG |  |
| SSA1_F | CTGCTGTTCAAGCTGCCATT | Primers to detect the *SSA1* gene expression involved in invasion in host cell and tissues |
| SSA1_R | CACCTGGTTGGTTATCGGCA |  |
| EAP1_F | TACTCCAGGCACTGAAGCTACTCC | Primers to detect the *EAP1* gene expression involved in adhesion and biofilm formation |
| EAP1_R | GAACACATCCACCTTCGGGACAG |  |
| HGC1_F | CAGCTTCCTGCACCTCATCAA | Primer to detect the *HGC1* gene expression involved in filamentous growth, biofilm formation and regulation of CDC28 kinase during hyphal growth |
| HGC1_R | AGCACGAGAACCAGCGATACT |  |
| CDC28_F | AAGGTGTACCTAGTACCGCCA | Primers to detect the *CDC28* gene expression involved in morphology-related transcription factors (especially hyphae-specific genes) |
| CDC28_R | RTAGCCCCTAGTCCAACTCCT |  |
| RAS1_F | GTGGTGGTGTTGGTAAATCCG | Primers to detect the *RAS1* gene expression involved in regulation of both a MAP kinase signaling pathway and a cAMP signaling pathway |
| RAS1_R | GTCCAGCAGTATCTAAAACATCCAA |  |
| TPK2_F | CCCAATCTGCTACCTCATCACTCC | Primers to detect the *TPK2* gene expression involved in regulation of cAMP signaling pathway |
| TPK2_R | AGGCAACAACGATCTTCTGATGGC |  |
| EFG1_F | TATGCCCCAGCAAACAACTG | Primers to detect the *EFG1* gene expression involved in adhesion, hyphal formation, invade and injure endothelial cells biofilm development |
| EFG1_R | TTGTTGTCCTGCTGTCTGTC |  |
| CPH1_F | ﻿GGCGGCAGTGATAGTGCAA | Primers to detect the *CPH1* gene expression involved in hyphal development |
| CPH1_R | ﻿GATTTTTCACTGCCATTACTTGGTT |  |
| UME6_F | TGGCTCCACTTACAAATCATAGT | Primers to detect the *UME6* gene expression involved in cell cycle progression, adhesion on the plastic surfaces, hyphal elongation and filamentous biofilms formation |
| UME6_R | GCTTGTTGTTGAGGTGGTAATG |  |
| CEK1_F | AACGCTGCAGCTACTACTTCTT | Primers to detect the *CEK1* gene expression involved in MAP kinase activity to regulate hypha formation and cell wall synthesis |
| CEK1_R | TGTTGTGATGGTTTATGAATGGCTG |  |
| ACS1_F | TTTCTCCGGTGATGGTGCTG | Primers to detect the *ACS1* gene expression, encoding acetyl-coenzyme A synthetase to regulate the glycolysis activity under hypoxic |
| ACS1_R | CATATGCAGCAACGGCTTGA |  |

**2.3 Schematic diagram of *SIR2* mutant strain construction**


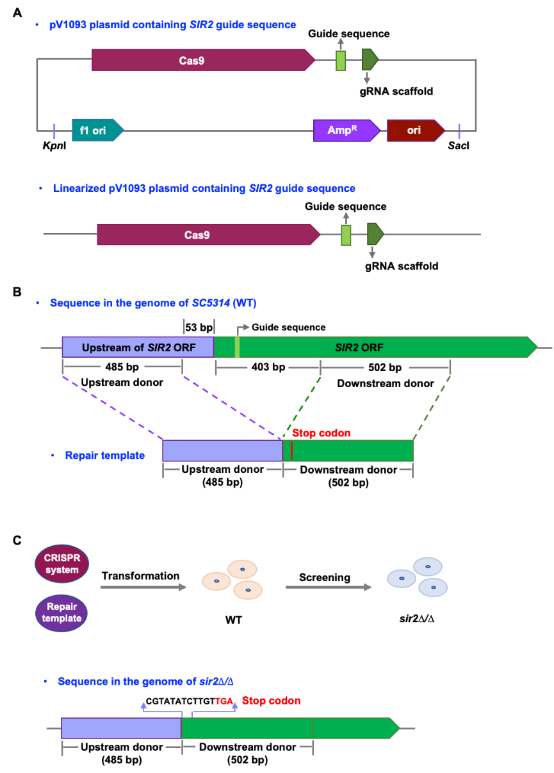


**Fig. S1 Schematic diagram of the construction of *sir2***$\boldsymbol{\Delta}$***/***$\boldsymbol{\Delta}$**.** **A.** Schematic diagram of CRISPR system containing the guide sequence to target *SIR2* gene, **B.** Design schematic of repair template containing stop codon that can blocks the transcription of *SIR2* gene, **C.** Construction schematic of *SIR2* deletion strains and the sequence schematic of *SIR2* locus in *sir2*$\Delta$*/*$\Delta$, TGA is the stop codon to block the transcription of retained *SIR2* gene.

**2.4 Schematic diagram of *SIR2* expression plasmid construction**


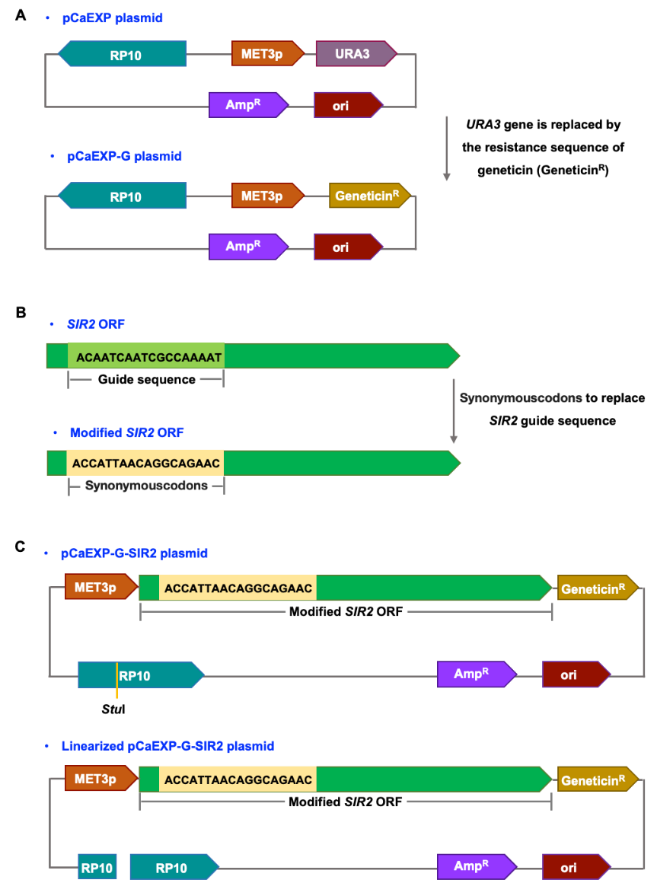


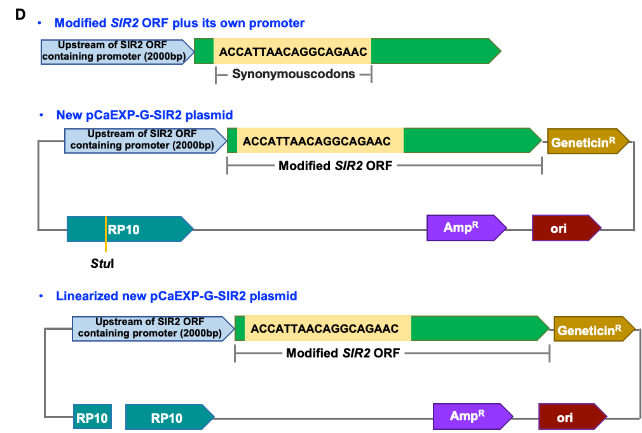


**Fig. S2 Schematic diagram of *SIR2* expression plasmid construction. A.** Schematic construction of modified pCaEXP plasmid (pCaEXP-G) that is created by replacing *UAR3* gene into the resistance sequence of geneticin, **B.** Schematic of modified *SIR2* ORF sequence that the SIR2 guide sequence is replaced by synonymouscodons, **C.** Schematic construction of *SIR2* expression plasmid (pCaEXP-G-SIR2) and *SIR2* gene expression cassette (Linearized pCaEXP-G-SIR2) that the pCaEXP-G-SIR2 is linearized at RP10 locus by digestion with *Stu*I. **D** Schematic construction of *SIR2* expression plasmid (new pCaEXP-G-SIR2) and *SIR2* gene expression cassette with its own promoter (Linearized new pCaEXP-G-SIR2) that the new pCaEXP-G-SIR2 is linearized at RP10 locus by digestion with *Stu*I.

**2.5 PCR verification and *SIR2* gene expression levels examination**


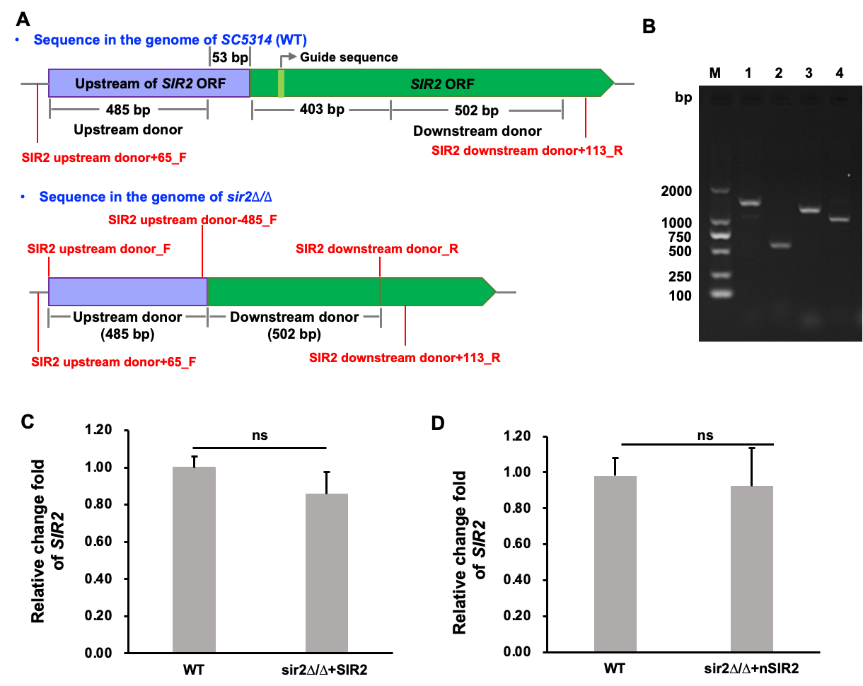

**Fig. S3 PCR verification of *SIR2* deletion and examination of *SIR2* expression levels. A.** Schematic diagram of primers to detect *SIR2* mutation, **B.** DNA gel of PCR products amplified by primers. M: DNA marker, 1: PCR product (1621 bp) amplified by primers (SIR2 upstream donor+65_F and SIR2 upstream donor+113_R) from genome of WT, 2: PCR product (641 bp) amplified by primers (SIR2 upstream donor-485_F and SIR2 upstream donor+113_R) from genome of *sir2*$\Delta$*/*$\Delta$, 3: PCR product (1165 bp) amplified by primers (*SIR2* upstream donor+65_F and SIR2 upstream donor+113_R) from genome of *sir2*$\Delta$*/*$\Delta$, 4: Repair template amplified by primers (SIR2 upstream donor_F and SIR2 downstream donor_R), **C.** Relative expression levels of *SIR2* gene in WT and *sir2*$\Delta$*/*$\Delta$+*SIR2*, detected by RT-qPCR (primers presented in Table S2); **D.** Relative expression levels of *SIR2* gene in WT and *sir2*$\Delta$*/*$\Delta$+n*SIR2*, detected by RT-qPCR (primers presented in Table S2); ns: no statistical difference; all the gene expression levels were determined in three biological replicates, each tested in triplicate.

**2.6 *In vivo* virulence of complemented strain expressing *SIR2* under its own promoter**


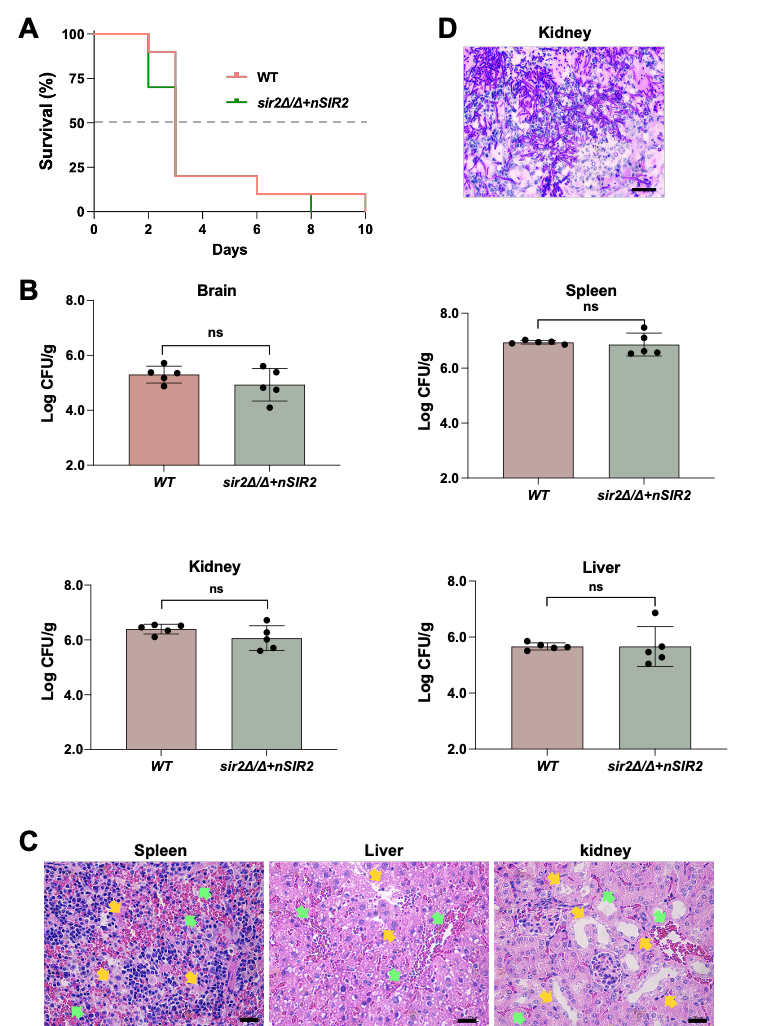


**Fig. S4 Virulence of *sir2***$\boldsymbol{\Delta}$***/***$\boldsymbol{\Delta}$***+nSIR2* strain in mice. A. ﻿**Animal survival assays. Mice (n=10) were intravenously inoculated with *sir2*$\Delta$*/*$\Delta$*+nSIR2* strain and were assessed for survival for up to 8 days post infection; wild-type *C. albicans* (WT) was as control and the data of virulence of WT came from Fig. 1 in manuscript. gray dotted line: median survival. **B. ﻿**Organ fungal burden assays. Mice were injected with *sir2*$\Delta$*/*$\Delta$*+nSIR2* strain and detected at 2 days post-infection; CFUs from brains, spleens, kidneys and livers were assayed and plotted using scatter diagram, with error bar; ns: no statistical difference based on unpaired t test (WT VS. *sir2*$\Delta$*/*$\Delta$*+nSIR2*). The data of virulence of WT came from Fig. 1 in manuscript **C.** Histopathological examination. Histopathological changes in spleens, livers and kidneys of mice were detected at 2 days post-infection by *sir2*$\Delta$*/*$\Delta$*+nSIR2* strain (hematoxylin and eosin (H&E) staining); WT was as control and the data of virulence of WT came from Fig. 1 in manuscript; All tissues tested showed severe bleeding and cellular structural damage, green arrow: hyperaemia, yellow arrow: cell structure damage, bar: 50 μm. **D.** Distribution of *C. albicans* in the kidneys of mice was detected at 2 days post-infection by *sir2*$\Delta$*/*$\Delta$*+nSIR2* strain (periodic acid-schiff (PAS) staining). WT was as control and the data of virulence of WT came from Fig. 1 in manuscript; Large of mycelia were observed in kidney tissues, bar: 50 μm.

**2.7 Hyphae frequencies**

**Fig. S5 Hyphae frequencies of various *C. albicans* at different time points cultured at 37℃ in 1640 medium containing 10% serum.**

**2.8 Hyphae formation and development**


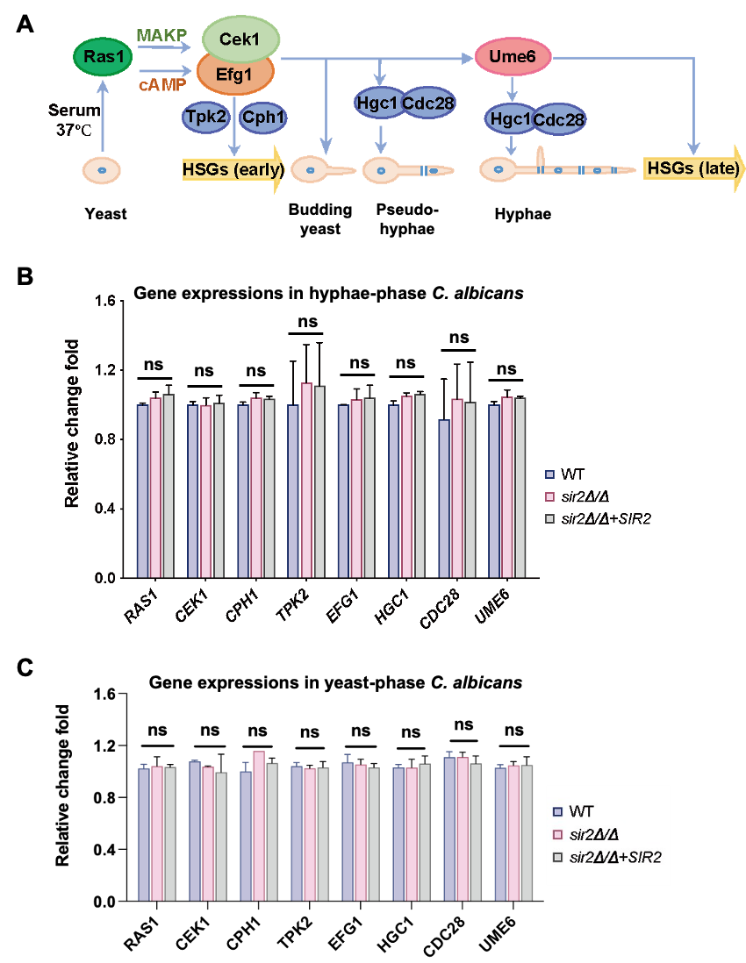


**Fig. S6 Relative expression levels of regulatory genes of hyphal formation and development in RPMI 1640 medium containing 10% serum at 37°C or in YPD medium at 30°C. A.** Schematic diagram of MAPK and cAMP/PKA: the mitogen-activated protein kinase (MAPK) pathway and the adenylate cyclase/protein kinase (cAMP/PKA) pathway are closely related to the hyphal formation and development of *C. albicans*; HSGs: hypha specific genes, **B.** Differential expression of genes involved in hyphal formation and development in hyphae-phase *C. albicans*. **C.** Differential expression of genes involved in hyphal formation and development in yeast-phase *C. albicans*. Data were collected in three independent replicate experiments. The mRNA levels were normalized on the basis of corresponding gene expression levels in yeast-form or hypha-form WT, and analyzed using the one-way ANOVA (WT VS. *sir2*$\Delta$*/*$\Delta$, WT VS. *sir2*$\Delta$*/*$\Delta$+*SIR2* and *sir2*$\Delta$*/*$\Delta$ VS. *sir2*$\Delta$*/*$\Delta$+*SIR2*); Average values plus error bars are shown in the histogram, ns: no statistical difference; all the gene expression levels were determined in three biological replicates, each tested in triplicate.

**2.9 Adhesion on host cells**

**
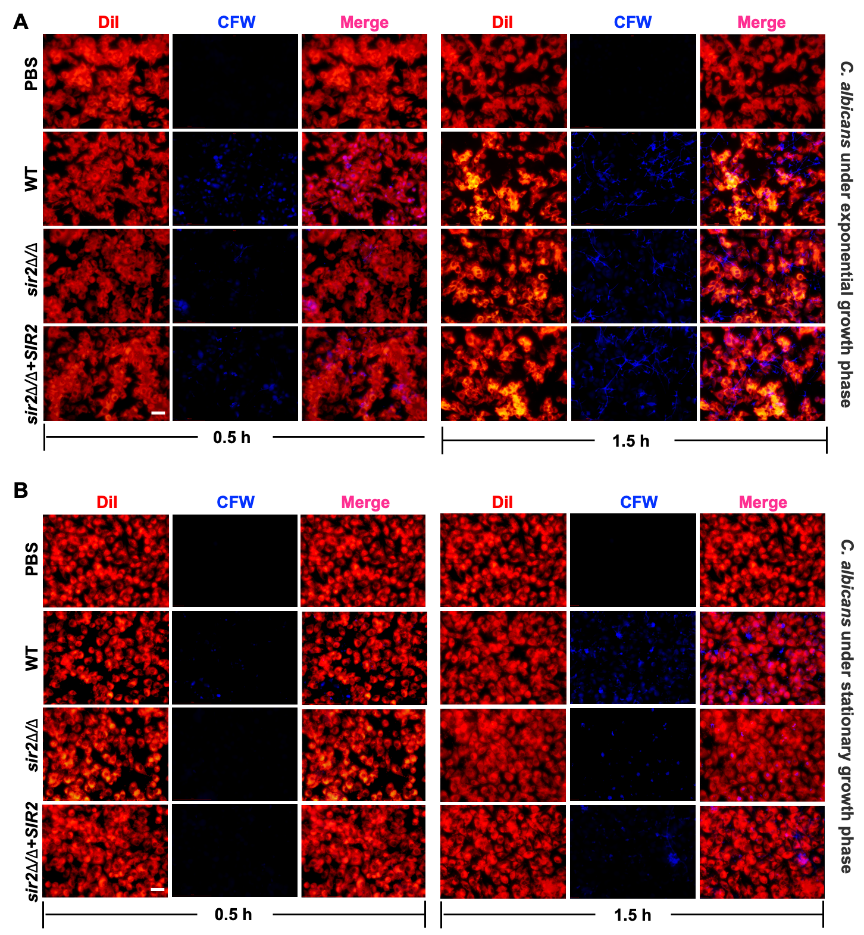
**

**Fig. S7 Adhesion ability of WT, *sir2***$\boldsymbol{\Delta}$***/***$\boldsymbol{\Delta}$ **and *sir2***$\boldsymbol{\Delta}$***/***$\boldsymbol{\Delta}$**+*SIR2* on L-929 cells.** **A.** Fluorescence photographs of adhesion of WT, *sir2*$\Delta$*/*$\Delta$ and *sir2*$\Delta$*/*$\Delta$+*SIR2* under exponential growth phase on L-929 cells at 0.5h and 1.5 h, bar: 100 μm, **B.** Fluorescence photographs of adhesion of WT, *sir2*$\Delta$*/*$\Delta$ and *sir2*$\Delta$*/*$\Delta$+*SIR2* under stationary growth phase on L-929 cells at 0.5h and 1.5 h, bar: 100 μm. Images are representative of two independent replicate experiments.

**2.10 Exposure of cell wall β-glucan**


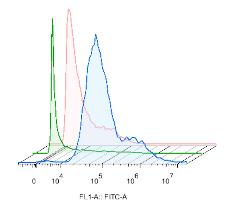

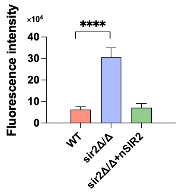


**β-glucan exposures**

WT

*sir2Δ/Δ*

*sir2Δ/Δ+nSIR2*

**Fig. S9**

**Fig. S8 Flow cytometry analysis of the exposures of cell wall β-glucan in WT, *sir2***$\boldsymbol{\Delta}$***/***$\boldsymbol{\Delta}$ **and *sir2***$\boldsymbol{\Delta}$***/***$\boldsymbol{\Delta}$**+*nSIR2* strains.** Histogramplots are representative of data collected in three independent replicate experiments (left); Measurements were analyzed using the one-way ANOVA (WT VS. *sir2*$\Delta$*/*$\Delta$, WT VS. *sir2*$\Delta$*/*$\Delta$+*SIR2* and *sir2*$\Delta$*/*$\Delta$ VS. *sir2*$\Delta$*/*$\Delta$+*SIR2*); Asterisks show statistically significant differences (^****^, *p*$<$0.0001) (right).

**2.11 Cell wall integrity**


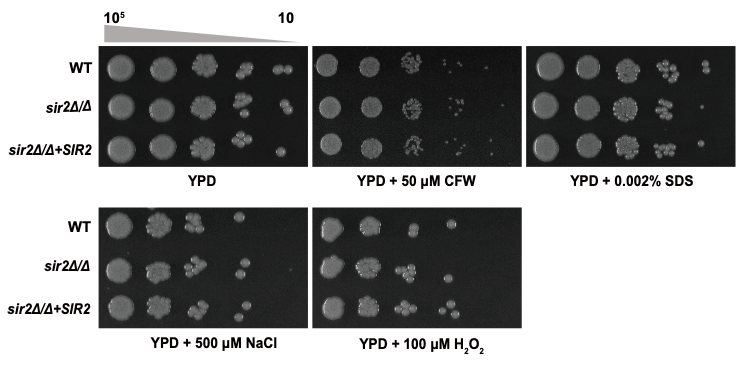


**Fig. S9 Susceptibility of different strains to various stressors.** Serial 10-fold dilutions of the indicated strains were used to inoculate YPD plates containing CFW (cell wall stress), SDS (plasma membrane stress), NaCl (osmotic stress) or H_2_O_2_ (oxidant stress) and incubated at 30℃ for 48 h. Images are representative of three independent replicate experiments; Images are representative of three independent replicate experiments.

**2.12 Alternative carbon utilization analyzed using spot dilution assay**

**Fig. S10 Carbon utilization capacity of different strains on YNB solid media.** Serial 10-fold dilutions of the indicated strains were used to inoculate YNB plates containing 2% glucose (m/v), 2% glycerol (v/v), 2% ethanol (v/v) or 2% potassium acetate (v/v) and incubated at 30℃ under normoxia or hypoxic condition for 2-4 days. Images are representative of three independent replicate experiments.

**3. References**

1. V. K. Vyas, M. I. Barrasa, G. R. Fink, A Candida albicans CRISPR system permits genetic engineering of essential genes and gene families. *Sci Adv* **1**, (2015).
